# Prognostic and diagnostic impact of fibrinogen, neutrophil-to-lymphocyte ratio, and platelet-to-lymphocyte ratio on thymic epithelial tumors outcome

**DOI:** 10.18632/oncotarget.25076

**Published:** 2018-04-24

**Authors:** Stefan Janik, Thomas Raunegger, Philipp Hacker, Bahil Ghanim, Elisa Einwallner, Leonhard Müllauer, Ana-Iris Schiefer, Julia Moser, Walter Klepetko, Hendrik Jan Ankersmit, Bernhard Moser

**Affiliations:** ^1^ Department of Thoracic Surgery, Division of Surgery, Medical University Vienna, Vienna, Austria; ^2^ Christian Doppler Laboratory for Diagnosis and Regeneration of Cardiac and Thoracic Diseases, Medical University Vienna, Vienna, Austria; ^3^ Department of Laboratory Medicine, Medical University Vienna, Vienna, Austria; ^4^ Clinical Institute of Pathology, Medical University Vienna, Vienna, Austria; ^5^ Departments of Dermatology and Venereology and Karl Landsteiner Institute of Dermatological Research, Karl Landsteiner University of Health Sciences, St. Pölten, Austria; ^6^ Head FFG Project “APOSEC“, FOLAB Surgery, Medical University Vienna, Vienna, Austria

**Keywords:** thymic epithelial tumors, thymoma, thymic carcinoma, fibrinogen, NLR

## Abstract

**Background:**

Peripheral blood-derived inflammation-based markers, such as Neutrophil-to-Lymphocyte Ratio (NLR), Platelet-to-Lymphocyte Ratio (PLR), and Fibrinogen have been identified as prognostic markers in various solid malignancies. Here we aimed to investigate the prognostic and diagnostic impact of NLR, PLR, and Fibrinogen in patients with thymic epithelial tumors (TETs).

**Results:**

Pretreatment Fibrinogen serum concentrations, NLRs and PLRs were highest in patients with TCs and advanced tumor stages. High pretreatment Fibrinogen serum concentration (≥452.5 mg/dL) was significantly associated with worse cause specific survival (CSS; *p* = 0.001) and freedom from recurrence (FFR; *p* = 0.043), high NLR (≥4.0) with worse FFR (*p* = 0.008), and high PLR (≥136.5) with worse CSS (*p* = 0.032). Longitudinal analysis revealed that compared to patients without tumor recurrence, patients with tumor recurrence had significantly higher NLR (11.8 ± 4.0 vs. 4.70 ± 0.5; *p* = 0.001) and PLR (410.8 ± 149.1 vs. 228.3 ± 23.7; *p* = 0.031).

**Conclusion:**

Overall, Fibrinogen serum concentrations, NLRs, and PLRs were associated with higher tumor stage, more aggressive tumor behavior, recurrence, and worse outcome. Prospective multicenter studies of the diagnostic and prognostic potential of Fibrinogen, NLR, and PLR are warranted.

**Methods:**

This retrospective analysis included 122 patients with TETs who underwent surgical resection between 1999-2015. Fibrinogen serum concentrations, NLRs, and PLRs were measured in patients preoperatively, postoperatively, and later during follow-up. These markers were analyzed for association with several clinical variables, including tumor stage, tumor subtype, FFR, and CSS and to evaluate their prognostic and diagnostic impact for detecting tumor recurrence.

## INTRODUCTION

Thymic epithelial tumors (TETs) are intrathoracic malignancies that, although rare, represent the most common anterior mediastinal tumors in adults. TETs show a unique association with the autoimmune disorder Myasthenia Gravis (MG). According to the WHO classification, TETs are histologically subdivided into A, AB, B1, B2, and B3 thymomas (and other rare subtypes) and thymic carcinomas (TCs) [1]. The Masaoka-Koga staging system has been used to pathologically grade TETs as stage I to IV depending on their level of invasiveness [2]. However, the International Thymic Malignancy Interest Group (ITMIG) recently proposed the use of the eighth edition of the TNM staging classification system for TETs by the American Joint Committee on Cancer (AJCC) and the Union for International Cancer Control (UICC), which might replace the currently accepted Masaoka-Koga staging system [3–6].

For advanced TETs, the mainstay of treatment is surgical tumor resection combined with multimodal therapy, which results in excellent 5- and 10-year overall survival (OS) rates of 96.5% and 90.9%, respectively [7, 8]. However, up to 30% of cases exhibit tumor recurrence, potentially even decades after initial therapy, necessitating lifelong follow-up mandatory [9, 10]. The lack of established and reliable biomarkers means that patients must undergo repeated chest computer tomography (CT) scans [8, 11]. Thus, there exists a need for affordable diagnostic and prognostic biomarkers.

We recently demonstrated that increased serum concentrations of C-reactive protein (CRP), heat shock proteins (HSPs), and high-mobility group box-1 (HMGB1) were associated with advanced tumor stage and worse outcome [7, 12, 13]. CRP monitoring is inexpensive, and CRP is currently assessed as a routinely measured marker for TET detection [14]. Each of the above-mentioned molecules is reportedly linked to inflammation and malignancies [15–17], with inflammatory responses playing crucial roles in various stages of tumor development, including tumor initiation, progression, and metastasis [18–21]. Peripheral blood-derived inflammation-based markers, such as CRP, Fibrinogen, the neutrophil-to-lymphocyte ratio (NLR), and the platelet-to-lymphocyte ratio (PLR) have been widely investigated and identified as prognostic markers in various solid tumors [22–25].

To date, only one study has investigated the roles of pretreatment NLR and PLR in patients with TCs, demonstrating that high pretreatment NLR was associated with larger tumor size and worse outcome [26]. However, no studies have examined the prognostic and diagnostic impact of NLR and PLR in thymomas, and their reliability in predicting tumor recurrence remains unknown. Furthermore, the role of Fibrinogen has not yet been elucidated in patients with TETs. In the present study, our primary aim was to assess the prognostic and diagnostic value of Fibrinogen serum concentration, NLR, and PLR in patients with TETs. We further evaluated whether lymphocytes, neutrophils, and platelets differed according to tumor subtype, tumor stage, or paraneoplastic MG. Finally, we evaluated the potential Fibrinogen expression in neoplastic thymic epithelial cells.

## RESULTS

### Patient cohort

For this study, we recruited a total of 122 patients, including 52 (42.6%) males and 70 (57.4%) females, with a mean age of 56.5 ± 16.1 years. Histological analysis revealed that the vast majority of patients presented with thymomas (*n =* 92; 75.4%), with the most common subtypes being B2 (19.7%) and AB (14.8%). The remaining 30 patients (24.6%) were diagnosed with TCs, with 29 patients having thymic squamous cell carcinomas (SCCs) and 1 having thymic adenocarcinoma of the enteric subtype. At time of diagnosis, 69.6% of patients presented with early Masaoka-Koga tumor stage (Stage I–II), with a mean tumor size of 58.5 ± 29.3 mm. Paraneoplastic MG was diagnosed in 26.2% of patients.

Among the cases, 45.9% were treated with surgical tumor resection alone, while 54.1% underwent multimodal treatment regimens combining surgery with radiotherapy and/or chemotherapy. Neoadjuvant therapy was performed in 18.8% of patients, and adjuvant therapy in 43.4%. Chemotherapy was mainly used in neoadjuvant settings, while radiotherapy was predominantly used in adjuvant settings. Radical tumor resection (R0) was achieved in 89.3% of cases.

### Preoperative Fibrinogen, NLR, and PLR in patients with TETs

Preoperative serum Fibrinogen, NLR, and PLR values were available in 112, 102, and 95 patients, respectively. Age and sex had no significant influence on Fibrinogen levels (*p =* 0.201 and *p =* 0.586, respectively), NLR (*p =* 0.888 and *p =* 0.419, respectively), or PLR (*p =* 0.715 and *p =* 0.976, respectively). Fibrinogen serum concentrations were significantly higher in patients with TETs (390.2 ± 11.4 mg/dL) compared to healthy volunteers (314.8 ± 10.7 mg/dL; *p <* 0.001). Moreover, Fibrinogen levels were significantly higher in patients with TCs (469.4 ± 30.9 mg/dL) compared to patients with thymomas (363.8 ± 9.7 mg/dL; *p =* 0.003). We also detected significantly elevated NLR and PLR in patients with TETs (3.4 ± 0.3 and 179.8 ± 12.1, respectively) compared to controls (1.8 ± 0.1 and 133.4 ± 7.1; *p <* 0.001 and *p =* 0.001, respectively). Similarly to Fibrinogen, the mean NLR and PLR values were significantly higher in patients with TCs (5.1 ± 0.8 and 268.2 ± 37.0, respectively) than in patients with thymomas (3.0 ± 0.2 and 154.8 ± 9.8; *p =* 0.020 and 0.007, respectively).

### Thymoma subtypes according to the WHO classification

According to WHO classification, Fibrinogen serum concentrations were lowest in patients with micronodular thymoma with lymphoid stroma (MNT) (331.7 ± 34.3 mg/dL) and those with AB thymomas (341.5 ± 26.0 mg/dL). NLR and PLR values were lowest in patients with B1 thymomas (2.19 ± 0.15 and 110.7 ± 15.0, respectively) and B2 thymomas (2.70 ± 0.4 and 124.5 ± 13.7, respectively; Table 1). Nonetheless, there were no significant differences between thymoma histotypes according to Fibrinogen serum concentrations (*p =* 0.418), NLR (*p =* 0.513) and PLR (*p =* 0.158), respectively.

**Table 1:** Preoperative analysis of Fibrinogen, NLR, and PLR in thymic epithelial tumors

Characteristics	*n*	Fibrinogen	*p-value*	*n*	NLR	*p-value*	*n*	PLR	*p-value*
		*mean (median) ± SD (SEM)*			*mean (median) ± SD (SEM)*			*mean (median) ± SD (SEM)*	
**TETs**	112	390.2 (360.5) ± 120.6 (11.4)		102	3.43 (2.64) ± 2.59 (0.3)		95	179.8 (142.4) ± 118.0 (12.1)	
**Controls**	27	314.8 (315.0) ± 56.5 (10.9)	<0.001^a^	51	1.78 (1.63) ± 0.75 (0.1)	<0.001^a^	48	133.4 (124.4) ± 49.4 (7.1)	0.001^a^
**Age (years)**									
<57	54	375.0 (328.5) ± 121.3 (16.5)		48	3.47 (2.49) ± 2.79 (0.40)		44	184.7 (150.0) ± 107.2 (16.2)	
≥57	58	404.3 (370.5) ± 119.3 (15.7)	0.201^a^	54	3.40 (2.86) ± 2.42 (0.33)	0.888^a^	51	175.7 (142.0) ± 127.4 (17.8)	0.715^a^
**Sex**									
Male	46	397.6 (369.5) ± 139.5 (20.6)		40	3.69 (2.92) ± 2.41 (0.38)		37	180.3 (138.7) ± 99.2 (16.3)	
Female	66	384.9 (353.0) ± 106.3 (13.1)	0.586^a^	62	3.27 (2.53) ± 2.71 (0.34)	0.419^a^	58	179.5 (150.0) ± 129.3 (17.0)	0.976^a^
**WHO**									
MNT	7	331.7 (327.0) ± 90.8 (34.3)		7	5.10 (2.42) ± 3.81 (1.44)		7	252.6 (274.4) ± 142.3 (53.8)	
A	14	405.2 (385.5) ± 82.0 (21.9)		14	3.32 (3.04) ± 2.04 (0.59)		12	198.8 (167.8) ± 89.6 (25.9)	
AB	16	341.5 (315.5) ± 104.1 (26.0)		16	2.93 (2.97) ± 0.82 (0.21)		14	140.7 (126.3) ± 59.0 (15.8)	
B1	10	361.9 (358.5) ± 54.8 (17.3)		10	2.19 (0.18) ± 0.49 (0.15)		10	110.7 (83.8) ± 47.3 (15.0)	
B2	22	364.7 (351.5) ± 87.8 (18.7)		22	2.70 (2.47) ± 1.89 (0.42)		18	124.5 (108.6) ± 58.1 (13.7)	
B3	15	363.5 (334.0) ± 96.9 (25.0)		15	2.71 (2.53) ± 1.47 (0.39)		13	152.4 (139.5) ± 71.3 (19.8)	
TC	28	469.4 (473.0) ± 163.4 (30.9)	0.003^b^	28	5.09 (4.05) ± 3.84 (0.82)	0.005^b^	21	268.2 (230.0) ± 169.6 (37.0)	<0.001^b^
**Tumor Stage I**									
I	25	354.5 (346.0) ± 65.1 (13.0)		25	2.84 (2.44) ± 1.69 (0.36)		20	171.3 (163.6) ± 74.4 (16.7)	
II	53	364.1 (352.0) ± 96.9 (13.3)		53	3.09 (2.69) ± 2.08 (0.29)		48	145.3 (129.4) ± 79.3 (11.5)	
III	11	428.1 (415.0) ± 126.5 (38.1)		11	4.74 (4.10) ± 3.88 (1.29)		9	268.9 (281.4) ± 145.5 (48.5)	
IV	23	470.8 (465.0) ± 169.3 (35.3)	0.001^b^	23	4.45 (3.22) ± 3.54 (0.81)	0.061^b^	18	236.9 (173.0) ± 179.7 (42.4)	0.003^b^
**Tumor Stage II**									
Early I-II	78	361.0 (349.5) ± 87.7 (9.93)		74	3.01 (2.61) ± 1.97 (0.23)		68	152.9 (135.6) ± 78.3 (9.49)	
Advanced III-IV	34	457.0 (463.0) ± 156.1 (26.8)	0.002^b^	28	4.54 (3.31) ± 3.58 (0.68)	0.040^b^	27	247.6 (177.1) ± 166.9 (32.1)	0.008^b^
**Myasthenia Gravis**									
Yes	31	351.8 (330.0) ± 102.3 (18.4)		31	3.11 (2.40) ± 2.75 (0.53)		26	161.5 (133.5) ± 148.4 (29.1)	
No	81	404.8 (368.0) ± 124.5 (13.8)	0.037^a^	81	3.55 (2.83) ± 2.54 (0.29)	0.455^a^	69	186.7 (158.8) ± 104.7 (12.6)	0.356^a^

Preoperative Fibrinogen serum values, NLR and PLR are shown with regards to sex, age, WHO classification, tumor stage and Myasthenia Gravis. Data are indicates as mean (median) ± SD (SEM). NLR, Neutrophil-to-Lymphocyte Ratio; PLR, Platelet-to-Lymphocyte Ratio; SD, standard deviation; SEM, standard error of the mean; TETs, Thymic Epithelial Tumors; WHO, World Health Organization classification of histologic tumor subtype. ^a^unpaired Student’s *t*-test; ^b^ one-way ANOVA.

### Masaoka-koga tumor stage

**Figure 1:**
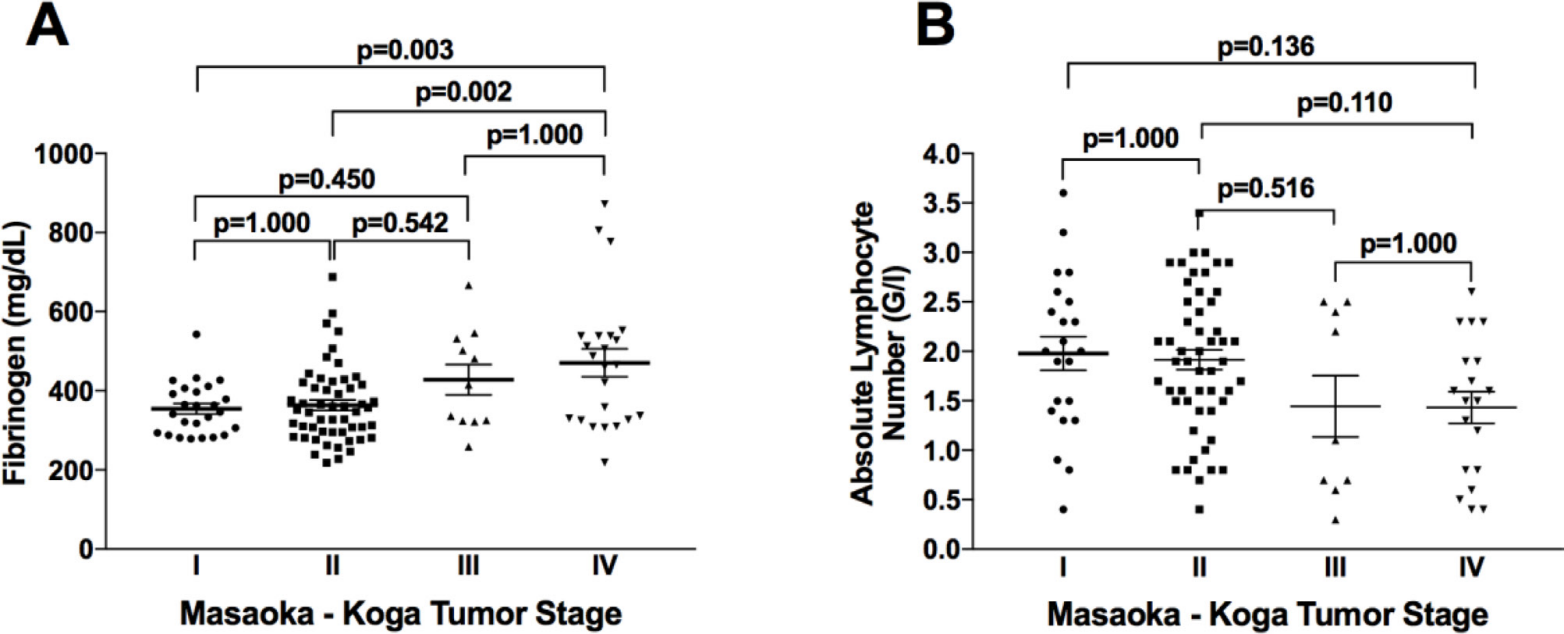
Fibrinogen and absolute lymphocyte numbers according to clinical Masaoka-Koga tumor stage Fibrinogen serum concentrations gradually increased with invasiveness level (**A**). Peripheral blood absolute lymphocyte numbers gradually decreased with tumor stage (**B**).

Compared to patients with early stage tumors (stage I–II), those with advanced stage tumors (stage III–IV) had significantly higher Fibrinogen levels (457.0 ± 26.8 mg/dL vs. 361.0 ± 9.9 mg/dL; *p =* 0.002), NLR (4.54 ± 0.7 vs. 3.01 ± 0.2; *p =* 0.040) and PLR (247.6 ± 32.1 vs. 152.9 ± 9.5; *p =* 0.008) (Table 1). Interestingly, Fibrinogen serum concentrations gradually increased with the level of invasiveness, with serum levels of 354.5 ± 13.0 mg/dL in non-invasive stage I tumors and 470.8 ± 35.3 mg/dL in stage IV TETs (Figure 1A).

Comparison of early stage thymomas (stages I–II) vs. advanced stage thymomas (stage III–IV) showed no significant differences in Fibrinogen serum concentrations (360.4 ± 10.3 mg/dL vs. 383.9 ± 29.3 mg/dL; *p =* 0.400), NLR (2.95 ± 0.22 vs. 3.17 ± 0.70; *p =* 0.730), or PLR (150.7 ± 10.1 vs. 177.9 ± 33.1; *p =* 0.328). Similarly, comparison of early stage TCs (stage I-II) vs. advanced stage TCs (stage III-IV) showed no significant differences in Fibrinogen (368.5 ± 41.3 mg/dL vs. 496.9 ± 35.8 mg/dL; *p =* 0.088), NLR (3.90 ± 1.44 vs. 5.43 ± 1.0; *p =* 0.447), or PLR (180.7 ± 23.7 vs. 295.5 ± 46.3; *p =* 0.194).

### Paraneoplastic MG

MG was diagnosed in 32 (26.2%) of the 122 patients, including in 45.8% of B2 thymomas (11 out of 24) and 41.2% of B3 thymomas (7 out of 17). Among these 32 patients, preoperative Fibrinogen, NLR, and PLR values were available for 31, 27, and 26, respectively. Compared to MG-positive patients, MG-negative patients had significantly higher preoperative Fibrinogen serum concentrations (404.9 ± 124.4 mg/dL vs. 351.8 ± 102.3 mg/dL; *p =* 0.037) and tumor size (6.1 ± 3.1 cm vs. 4.5 ± 2.9 cm; *p =* 0.049). However, after exclusion of TCs, the difference in Fibrinogen serum concentration between MG-negative and MG-positive thymomas was not significant (377.5 ± 13.0 mg/dL vs. 337.7 ± 12.5 mg/dL; *p =* 0.051). NLR and PLR did not significantly differ according to MG status (*p =* 0.455 and *p =* 0.356, respectively).

The majority of MG patients (63.2%) received therapy with acetylcholinesterase inhibitors as single therapy or in combination with azathioprine (21.1%) or corticosteroids (26.4%). Myasthenic patients treated with acetylcholinesterase inhibitors alone compared to those treated with additive immunosuppressive regimens did not significantly differ in Fibrinogen level (342.7 ± 75.5 mg/dL vs. 330.4 ± 76.7 mg/dL; *p =* 0.739), NLR (1.96 ± 0.8 vs. 4.38 ± 3.2; *p =* 0.164), or PLR (105.0 ± 28.3 vs. 165.5 ± 87.9; *p =* 0.052).

### Neutrophil, lymphocyte, and platelet blood counts in patients with TETs

We next evaluated how neutrophil, lymphocyte, and platelet blood counts were associated with clinical characteristics, including WHO classification, Masaoka-Koga tumor stage, and paraneoplastic MG. Absolute and relative neutrophil and lymphocyte counts and absolute platelet counts did not significantly differ according to WHO classification or Masaoka-Koga tumor stage (Table 2). Although this difference was not significant, absolute lymphocyte numbers gradually decreased with level of invasiveness, from 1.98 ± 0.80 G/L at non-invasive stage I to 1.43 ± 0.70 G/L at metastasized stage IV (*p =* 0.822; Figure 1B). We observed the highest lymphocyte levels in patients with B1 and B2 thymomas, and the lowest levels in TCs. Absolute lymphocyte numbers were significantly higher in patients with paraneoplastic MG (2.09 ± 0.7 G/L) compared to MG-negative patients (1.69 ± 0.1 G/L; *p =* 0.020), while neutrophil, platelet, and relative lymphocyte numbers did not significantly differ between these two patient groups (Table 2).

**Table 2:** Neutrophil, lymphocyte, and platelet numbers according to clinical characteristics of patients with TETs

	Neutrophils		Lymphocytes		Platelets	
	*abs. (rel.)*	*p-value*	*abs. (rel.)*	*p-value*	*abs.*	*p-value*
**WHO**						
MNT	4.53 (70.40%)		1.34 (20.6%)		252.3	
A	3.19 (64.6%)		1.53 (24.0%)		286.5	
AB	4.52 (66.3%)		1.82 (24.2%)		243.7	
B1	4.73 (60.2%)		2.35 (31.1%)		235.0	
B2	4.32 (62.4%)		2.23 (28.7%)		272.1	
B3	3.78 (62.6%)		1.96 (27.5%)		280.9	
TC	3.50 (68.6%)	0.820 (0.230)^a^	1.30 (19.8%)	0.958 (0.065)^a^	279.1	0.166^a^
**Tumor Stage**						
I	3.70 (63.6%)		1.98 (27.1%)		299.5	
II	4.47 (64.3%)		1.92 (25.8%)		249.7	
III	3.49 (67.5%)		1.44 (21.1%)		266.8	
IV	3.43 (67.2%)	0.986 (0.262)^a^	1.43 (22.6%)	0.822 (0.076)^a^	277.1	0.676^a^
**MG**						
Yes	4.25 (63.4%)		2.09 (27.1%)		273.4	
No	3.89 (65.5%)	0.458 (0.352)^b^	1.69 (24.3%)	0.020 (0.200)^b^	266.5	0.753^b^

Data are presented as mean absolute (abs.; G/L) and relative (rel.; %) numbers of neutrophils and lymphocytes, and absolute (abs., G/L) number of platelets. TETs, Thymic epithelial tumors; WHO, World Health Organization classification of histologic tumor subtype; MG, Myasthenia Gravis; MNT, micronodular thymoma; TC, thymic carcinoma. ^a^ One-way ANOVA; ^b^ unpaired Student’s *t*-test.

### Prognostic analysis: survival and recurrence

**Figure 2:**
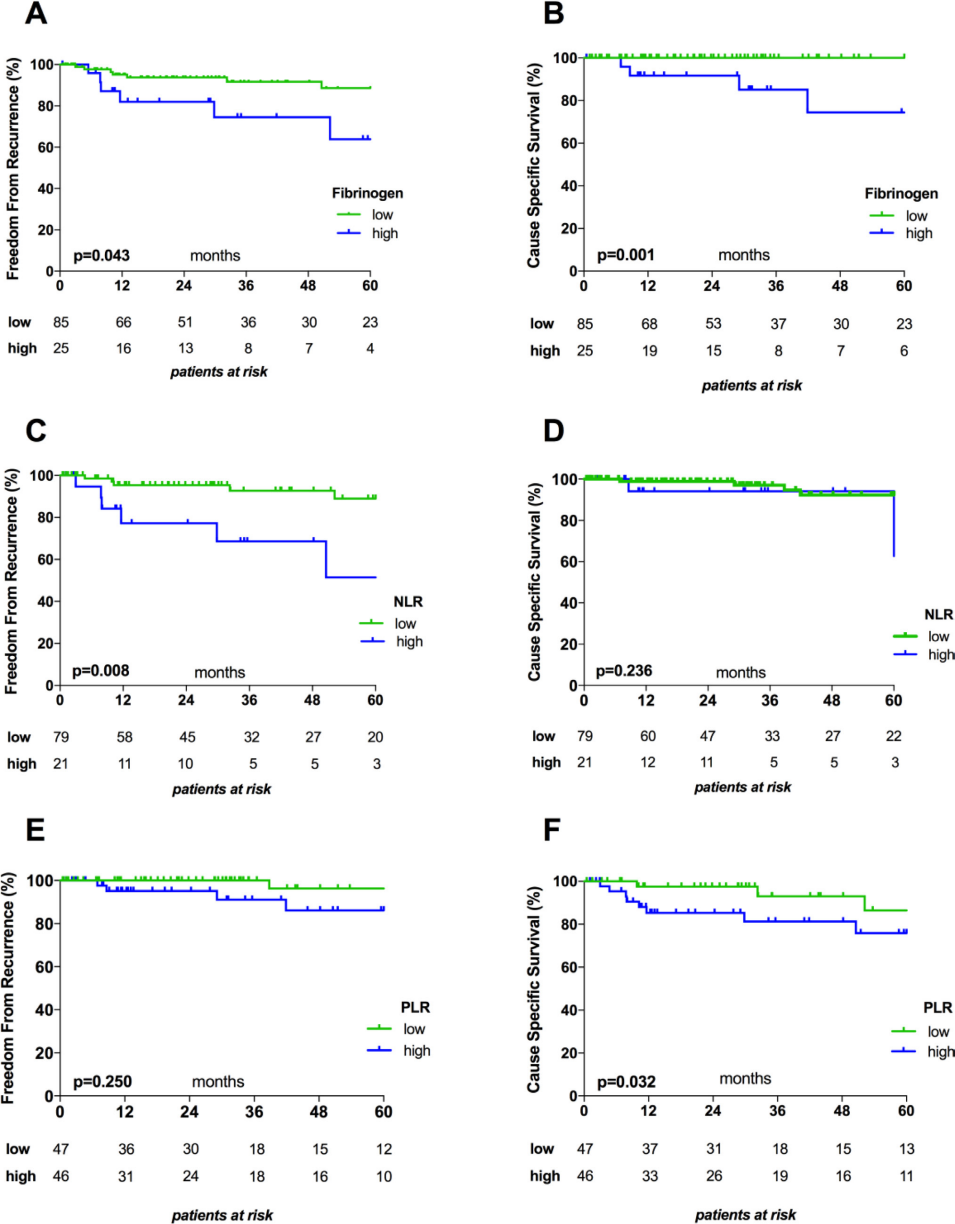
Kaplan–Meier survival curves for Fibrinogen, NLR, and PLR Graphs show the associations between Fibrinogen and FFR (**A**) and CSS (**B**), between NLR and FFR (**C**) and CSS (**D**) , and between PLR and FFR (**E**) and CSS (**F**). The cut-off values used to dichotomize patients into low and high subgroups were 452.5 mg/dL for Fibrinogen, 4.0 for NLR, and 136.5 for PLR. NLR, Neutrophil-to-Lymphocyte Ratio; PLR, Platelet-to-Lymphocyte Ratio.

Median follow-up time was 30.8 months (mean, 40.8 months). Of the 122 patients, 15 (12.3%) experienced recurrence, including 3 local (2.5%), 4 regional (3.3%), and 8 distant (6.6%) recurrences. We performed survival analyses to determine how freedom from recurrence (FFR) and cause specific survival (CSS) were impacted by pretreatment serum Fibrinogen concentration, NLR, and PLR. By calculating the Youden Index, we identified 452.5 mg/dL as an optimal cut-off value for discriminating between low and high Fibrinogen. To dichotomize patients into low and high NLR and PLR subgroups, we used an empiric cut-off value of 4.0 and a median of 136.5, respectively. High Fibrinogen was associated with significantly worse FFR (*p =* 0.043) and CSS (*p =* 0.001). Patients with high NLR showed significantly worse FFR (*p =* 0.008), while patients with high PLR showed significantly worse CSS (*p =* 0.032; Figure 2).

In separate analyses of thymomas and TCs, we found that high pretreatment Fibrinogen serum levels were associated with significantly worse CSS in patients with thymomas (*p =* 0.013), but not in patients with TCs (*p =* 0.119). On the other hand, FFR did not significantly differ in relation to Fibrinogen in either subgroup (*p =* 0.880 and *p =* 0.696). Pretreatment NLR and PLR values did not significantly affect CSS or FFR in patients with thymomas (CSS: *p =* 0.717 and *p =* 0.202, respectively; FFR: *p =* 0.572 and *p =* 0.792, respectively) or in patients with TCs (CSS: *p =* 0.506 and *p =* 0.526, respectively; FFR: *p =* 0.374 and *p =* 0.967, respectively).

We additionally performed univariable and multivariable Cox-regression analyses to assess the prognostic power of clinical characteristics, including histology (TC vs. thymoma), Masaoka-Koga tumor stage (early stage I-II vs. advanced stage III–IV), Fibrinogen (high vs. low), NLR (high vs. low), and PLR (high vs. low), with regards to FFR and CSS. Univariable analysis revealed that significantly worse FFR was associated with presence of TC (HR 4.93; *p =* 0.002) and high pretreatment NLR (HR 3.95; *p =* 0.014) but not with Masaoka-Koga tumor stage, PLR, or Fibrinogen. In multivariable analysis, only diagnosis of TC remained a significant and strong predictor of worse FFR (HR 8.55; *p =* 0.036). Conversely, worse CSS was associated with TC (HR 23.3; *p =* 0.004), advanced tumor stage (HR 12.35; *p =* 0.022) and high Fibrinogen (HR 17.24; *p =* 0.012) but not with high PLR or high NLR. None of the tested variables remained a significant prognostic factor for CSS in multivariable analysis (Table 3).

**Table 3:** Univariable and multivariable Cox regression analysis

	Univariable			Multivariable		
	HR	*p*-value	95% CI	HR	*p*-value	95% CI
**Freedom from recurrence**						
Histology (TC vs. Thymoma)	4.93	0.002	1.76–13.7	8.55	0.036	1.14–62.5
Tumor Stage (I–II vs. III–IV)	2.60	0.066	0.94–7.14	0.68	0.673	0.12–4.02
Fibrinogen (high vs. low)	2.86	0.053	0.99–8.26	1.40	0.601	0.40–4.95
NLR (high vs. low)	3.95	0.014	1.32–11.7	3.14	0.145	0.67–14.7
PLR (high vs. low)	2.00	0.259	0.60–6.67	0.44	0.350	0.08–2.46
**Cause specific survival**						
Histology (TC vs. Thymoma)	23.3	0.004	2.68–200.0	3.56	0.442	0.14–90.9
Tumor Stage (I-II vs. III-IV)	12.35	0.022	1.43–111.1	3.10	0.459	0.16–62.5
Fibrinogen (high vs. low)	17.24	0.012	1.87–166.7	9.09	0.149	0.45–166.7
NLR (high vs. low)	62.5	0.265	0.04–100.0	0.61	0.677	0.06–6.10
PLR (high vs. low)	2.83	0.257	0.47–16.5	1.07	0.963	1.00–1.60

Presented data indicate the prognostic impact of tested factors. Patients were dichotomized into high and low subgroups using cut-off values of 452.5 mg/dL for Fibrinogen, 4.0 for NLR, and 136.5 for PLR. TC, Thymic carcinoma; HR, Hazard Ratio; CI, Confidence Interval; NLR, Neutrophil-to-Lymphocyte Ratio; PLR, Platelet-to-Lymphocyte Ratio.

### Longitudinal analysis

**Figure 3:**
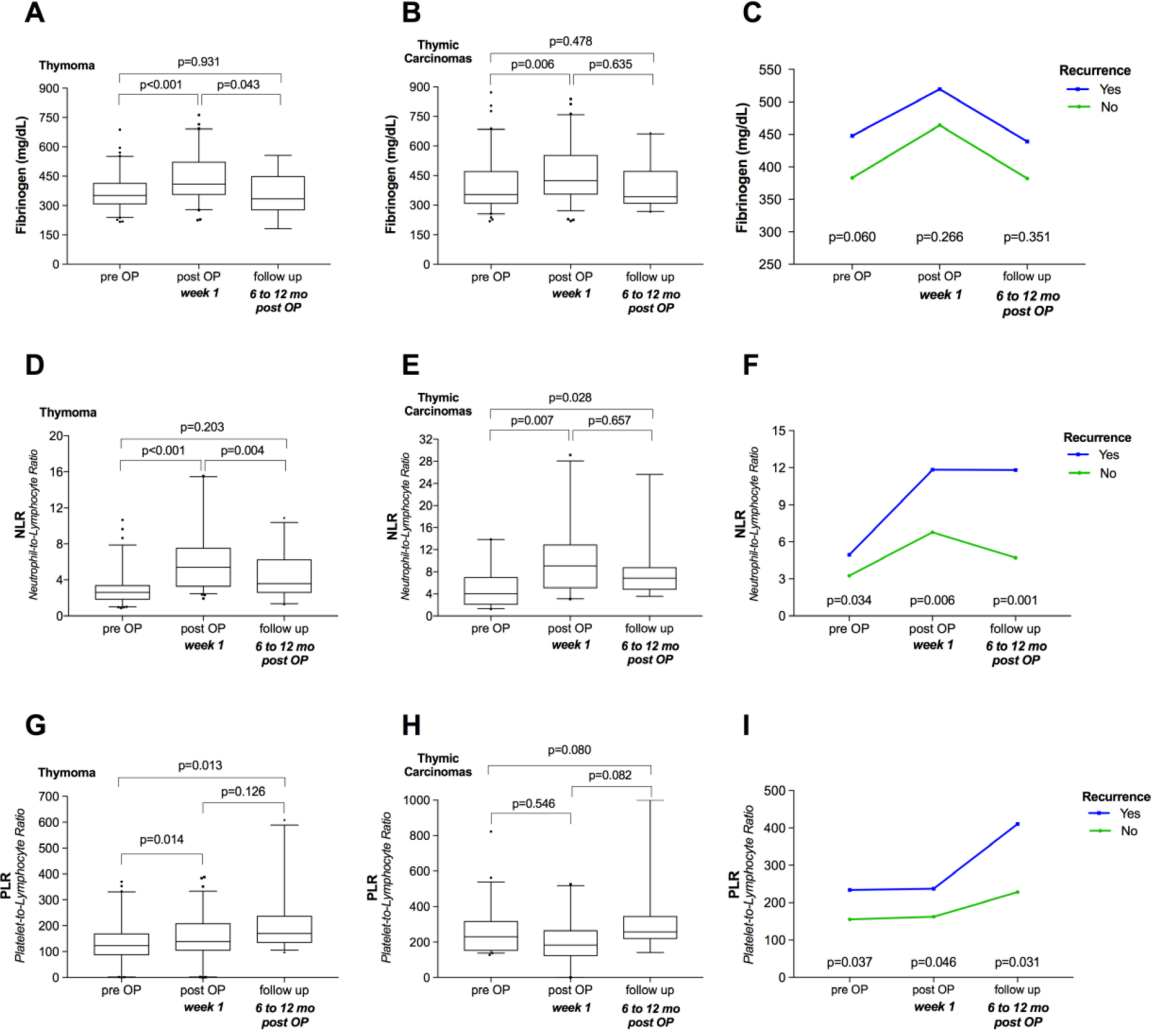
Longitudinal analysis of Fibrinogen, NLR, and PLR Plots illustrate how surgical tumor resection and recurrence were related to Fibrinogen, NLR, and PLR in thymomas (**A**, **D**, **G**) and thymic carcinomas (**B**, **E**, **H**), as well as the longitudinal courses of Fibrinogen, NLR, and PLR according to tumor recurrence among TETs (**C**, **F**, **I**). NLR, Neutrophil-to-Lymphocyte Ratio; PLR, Platelet-to-Lymphocyte Ratio; mo, months; TETs, thymic epithelial tumors.

Next, we performed longitudinal analysis of the preoperative, postoperative (3 to 7 days after surgery) and follow-up (6 to 12 months after surgery) measurements of Fibrinogen serum concentration, NLR, and PLR to evaluate the reliability of these parameters as tumor markers within oncologic follow-up. Compared to healthy controls, patients with TETs had significantly higher mean preoperative Fibrinogen serum levels (390.2 ± 11.4 mg/dL vs. 314.8 ± 10.9 mg/dL; *p <* 0.001), NLR (3.43 ± 0.3 vs. 1.78 ± 0.1; *p =* 0.001), and PLR (179.8 ± 12.1 vs. 133.4 ± 7.1; *p =* 0.001). Relative to the preoperative values, postoperative Fibrinogen serum concentrations and NLR values were significantly elevated in thymomas (Fibrinogen: 445.6 ± 14.7 mg/dL vs. 364.7 ± 9.8 mg/dL; *p <* 0.001; NLR: 6.22 ± 0.5 vs. 2.99 ± 0.2; *p <* 0.001) and in TCs (Fibrinogen: 541.5 ± 34.0 mg/dL vs. 469.4 ± 30.9 mg/dL; *p =* 0.006; NLR: 10.5 ± 1.4 vs. 5.1 ± 0.8; *p =* 0.007). At 6–12 months after tumor resection, Fibrinogen and NLR were significantly decreased compared to postoperative values in thymomas (Fibrinogen: 356.5 ± 25.2 mg/dL vs. 445.6 ± 14.7 mg/dL; *p =* 0.043; NLR: 4.37 ± 0.5 vs. 6.22 ± 0.5; *p =* 0.004) but were not significantly reduced in TCs (Fibrinogen: 454.3 ± 40.5 mg/dL vs. 541.5 ± 34.0 mg/dL; *p =* 0.635; NLR: 8.7 ± 2.0 vs. 10.5 ± 1.4; *p =* 0.657). In patients with TCs, follow-up NLR values were significantly higher than preoperative values (8.7 ± 2.0 vs. 5.1 ± 0.8; *p =* 0.028), while Fibrinogen levels did not significantly differ (454.3 ± 40.5 mg/dL vs. 469.4 ± 30.9 mg/dL; *p =* 0.478). In thymoma patients, follow-up Fibrinogen levels and NLR values were similar to preoperative values (Fibrinogen: 356.5 ± 25.2 mg/dL vs. 364.7 ± 9.8 mg/dL; *p =* 0.931; NLR: 4.37 ± 0.5 vs. 2.99 ± 0.2; *p =* 0.203) (Figure 3A, 3B, 3D, 3E).

We found divergent trends for PLR values in thymomas and TCs. Compared to preoperative values, postoperative PLR was significantly elevated in patients with thymomas (141.4 ± 9.8 vs. 156.9 ± 10.1; *p =* 0.014) but was decreased in patients with TCs (207.8 ± 29.3 vs. 168.2 ± 37.0; *p =* 0.546). Nonetheless, we detected the highest PLR values for thymomas (212.5 ± 24.8) and TCs (347.1 ± 74.0) during oncologic follow-up (Figure 3G, 3H).

Interestingly, separate analyses of patients with and without tumor recurrence revealed that patients with tumor recurrence showed significantly higher NLR (*p =* 0.001) and PLR (*p =* 0.031) at 6–12 months post-resection (Figure 3F, 3I). In particular, compared to patients without tumor recurrence, patients with tumor recurrence showed significantly lower absolute and relative lymphocyte numbers (*p =* 0.014 and *p =* 0.027, respectively), while platelet and neutrophil counts were slightly but not significantly higher (*p =* 0.492 and *p =* 0.154, respectively). In contrast, Fibrinogen serum concentrations did not significantly differ in patients with recurrence compared to those without (*p =* 0.351; Figure 3C).

### NLR and PLR as prognostic markers during oncologic follow-up

**Figure 4:**
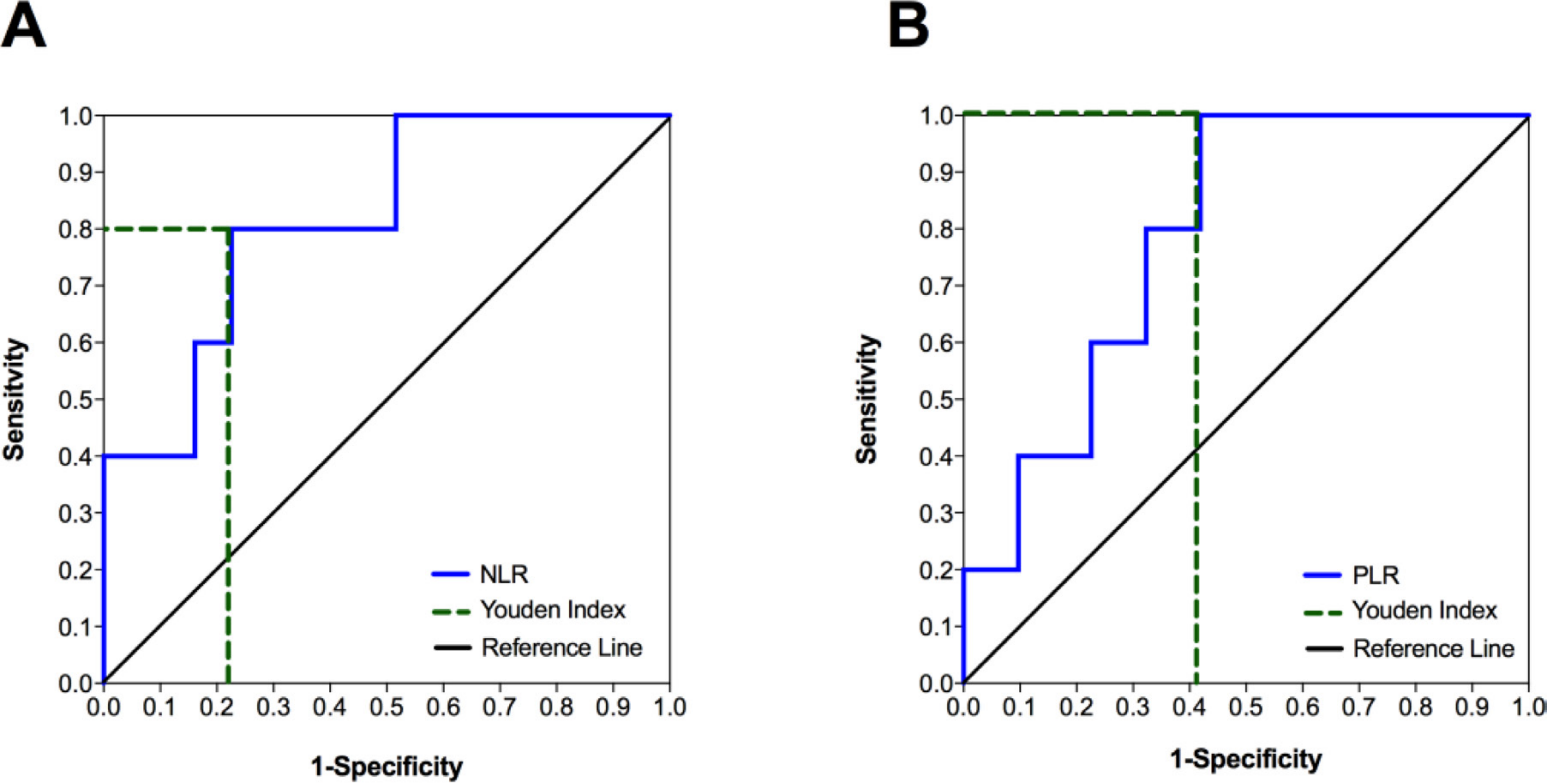
The accuracy of predicting tumor recurrence in patients with TETs based on NLR and PLR values during oncologic follow-up Receiver operating characteristic (ROC) curves for the use of NLR (**A**) and PLR (**B**) to predict tumor recurrence during oncologic follow-up showed area under the curve (AUC) values of 0.819 (*p* = 0.024) and 0.787 (*p* = 0.042), respectively. The dotted lines indicate the highest Youden Indices for NLR (Youden Index = 0.574; sensitivity = 0.800; specificity = 0.226; cut-off at 6.6) and PLR (Youden Index = 0.581; sensitivity = 1.000; specificity = 0.419; cut-off at 202.5). NLR, Neutrophil-to-Lymphocyte Ratio; PLR, Platelet-to-Lymphocyte Ratio.

We further evaluated the reliability of NLR and PLR as markers for the prediction of tumor recurrence during oncologic follow-up. We performed ROC analysis of both markers for predicting tumor recurrence (Figure 4). The area under the curve (AUC) was 0.819 for NLR and 0.787 for PLR, indicating that both markers had high accuracy to predict tumor recurrence.

Next, we calculated Youden Indices to define the optimal NLR and PLR cut-off values for predicting tumor recurrence. The highest Youden Indices (0.574 and 0.581, respectively) were found at cut-off values of 6.6 for NLR and 202.5 for PLR. Using a NLR value of 6.6 as a cut-off to predict tumor recurrence, we achieved a sensitivity of 80%, specificity of 77.4%, positive predictive value (PPV) of 36.4, and negative predictive value (NPV) of 96%. Using a PLR value of 202.5 as a cut-off, we achieved a sensitivity of 100%, specificity of 58.1%, PPV of 27.8%, and NPV of 100%.

Binary logistic regression analysis revealed that NLR was a significant factor for predicting tumor recurrence (*p =* 0.043; R^2^: 0.378), while PLR (*p =* 0.078; R^2^: 0.165) and Fibrinogen (*p =* 0.341; R^2^: 0.054) were not. Moreover, we did not find an additive effect regarding prediction of tumor recurrence when using combinations of NLR and PLR (NLR: *p =* 0.046; PLR: *p =* 0.847; R^2^: 0.379), NLR and Fibrinogen (NLR: *p =* 0.071; Fibrinogen: *p =* 0.913; R^2^: 0.369), or PLR and Fibrinogen (PLR: *p =* 0.165; Fibrinogen: *p =* 0.892; R^2^: 0.192).

### Fibrinogen expression in TETs

**Figure 5:**
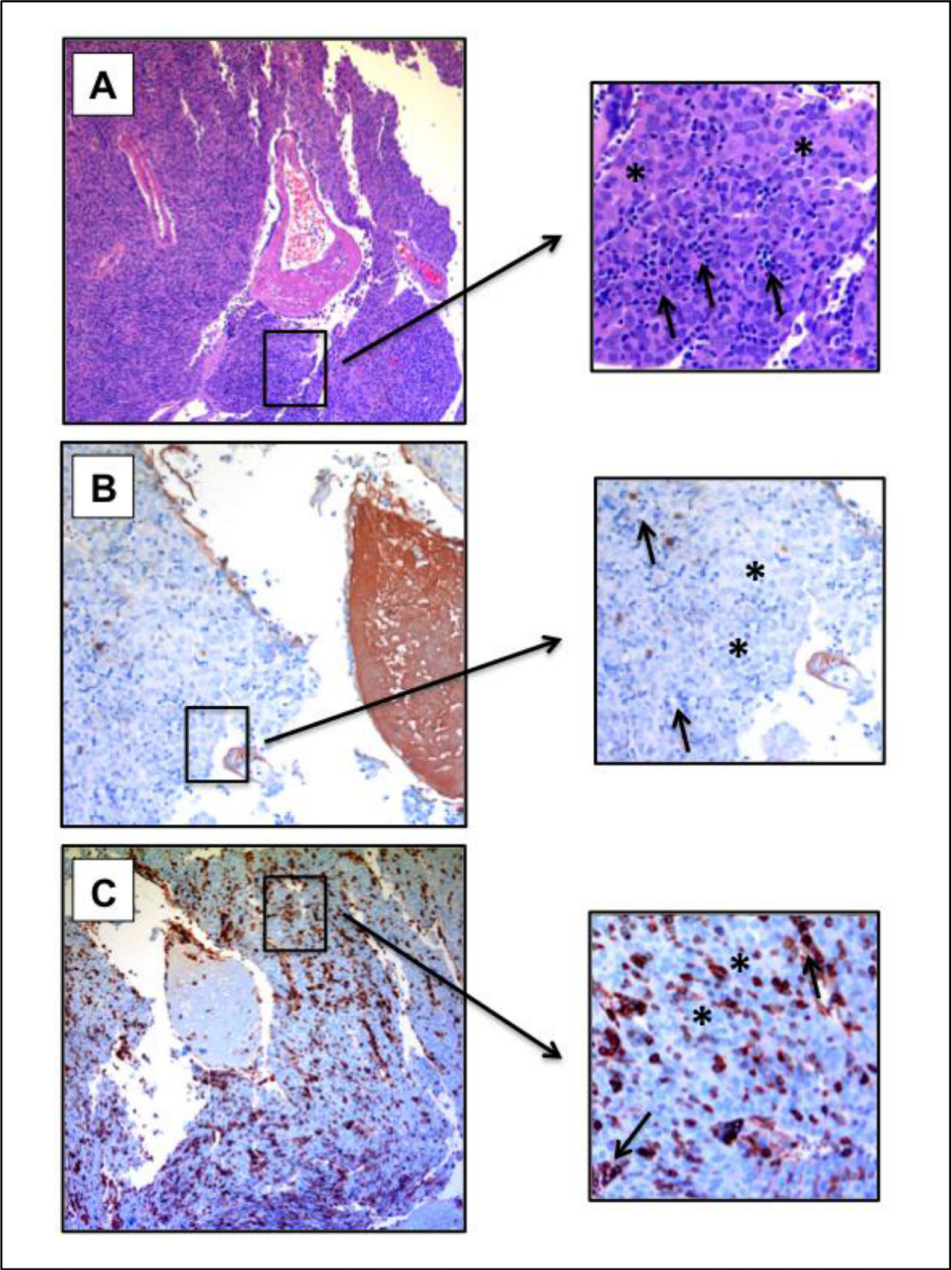
Fibrinogen expression in B2/B3 thymoma Staining of a B2/B3 thymoma (B3 part) with Hematoxylin-Eosin (**A**) and for Fibrinogen (**B**) and CD45 (**C**) expression. 100× magnification. Asterisks indicate neoplastic thymic epithelial cells. Arrows indicate cells of the hematopoietic lineage. Fibrinogen expression is absent from tumor cells and lymphocytes. Lymphocytes exhibit CD45 expression.

Finally, we performed immunohistochemical staining for Fibrinogen in representative B2/B3 thymomas to evaluate whether malignant thymic epithelial cells expressed Fibrinogen. Fibrinogen expression was detected on endothelial cells and within thrombotic clots, but was absent from neoplastic epithelial cells and cells of the hematopoietic lineage (Figure 5).

## DISCUSSION

Chronic inflammation plays a pivotal role in carcinogenesis, and accumulating evidence indicates that systemic biomarkers that are upregulated in response to cancer-related inflammation (including CRP, Fibrinogen, NLR, and PLR) potentially have diagnostic and prognostic power in various malignancies. Here we investigated the possible prognostic and diagnostic impact of Fibrinogen, NLR, and PLR in patients with TETs.

The clinical and demographic characteristics of our study population, including female-to-male ratio, age, and percentages of TCs and MG, were comparable to those in previous publications [9, 11, 27–29]. Within our cohort, high pretreatment Fibrinogen serum concentrations were associated with advanced tumor stage, and worse FFR and CSS. Notably, pretreatment Fibrinogen serum concentrations gradually increased from stage I to stage IV TETs, underlining the strong association between Fibrinogen and tumor invasiveness. With regards to WHO classification, Fibrinogen serum levels were highest in TCs, and lowest in AB thymomas and MNT. TCs are characterized by more aggressive tumor behavior, higher recurrence rates, and worse outcome, while AB thymomas and MNT are associated with a more indolent course and favorable outcome [8, 9, 30]. Similar associations between elevated pretreatment Fibrinogen serum levels and advanced disease stage and worse outcome have been reported in various other malignancies, including nasopharyngeal carcinoma, malignant pleura mesothelioma, ovarian cancer, endometrial cancer, and lymphomas [31–35].

Fibrinogen is an acute phase protein that is produced by the liver in response to proinflammatory cytokines [36, 37]. It is unclear whether increased Fibrinogen in advanced stage tumors is a bystander effect resulting from an enhanced immune response against higher tumor load, or if Fibrinogen plays an active role in tumor progression via promotion of tumor growth, angiogenesis, and metastasis [38–40]. Here we demonstrated that Fibrinogen staining was absent in malignant thymic epithelial cells, and that Fibrinogen serum concentrations gradually increased with rising invasiveness. These findings indicate that elevated Fibrinogen serum levels are likely a bystander effect of the immune response.

Similar to Fibrinogen, NLR and PLR were found to be highest in patients with TCs and advanced stage tumors that were associated with unfavorable outcome. These results are in line with the literature showing that high pretreatment NLR (>4.1) is associated with larger tumor size, higher Masaoka-Koga tumor stage, and decreased FFR and OS in patients with TCs [26]. Moreover, associations between high PLR and NLR and worse prognosis are reported in several malignancies, including paranasal sinus carcinomas, laryngeal SCC, esophageal carcinoma, colorectal cancer, and lymphomas [22, 41–46]. The majority of these studies report that NLR has superior prognostic impact compared to PLR, which is in accordance with our present findings that only NLR was a significant factor associated with worse CSS. However, no previously reported data indicate that NLR, PLR, Fibrinogen, or CRP can be used in a clinically meaningful way to help differentiate among anterior mediastinal tumors (TETs, lymphomas, and germ cell tumors) or to facilitate TET diagnosis.

It is not yet fully understood why increased NLR and PLR correspond with advanced stage tumors. High NLR and PLR imply increased neutrophils and platelets and/or decreased lymphocytes. In established tumors, the balance between inflammation-promoted tumor growth and anti-tumor immunity is tilted towards pro-tumor inflammation and tumor progression [18]. Pro-tumor inflammation is triggered by tumor and immune cells within the tumor microenvironment, resulting in suppression of lymphocytes or natural killer cells and increased recruitment of neutrophils or platelets [47–49]. Moreover, absolute lymphocyte counts gradually decrease from non-invasive to metastasized TETs, with the lowest lymphocyte numbers in patients with TCs. Although not all lymphocyte subsets are endowed with anti-tumor activity, lymphocytes are key players in tumor-specific immune responses [50]. Numerous studies demonstrate a favorable relationship between the amount of tumor-infiltrating lymphocytes (TILs) and outcome in various solid tumors, including oropharyngeal, nasopharyngeal, and esophageal SCC [51–54].

Paraneoplastic MG is characterized by muscle weakness caused by autoantibodies directed against peptides of the neuromuscular junction. Here we found significantly higher absolute lymphocyte counts in tumor patients diagnosed with MG. Similarly, Buckley *et al.* (2001) and Ströbel *et al.* (2002) reported significantly higher numbers of naïve CD4^+^ and CD8^+^ T cells in blood samples from patients with TETs and paraneoplastic MG [55, 56]. Thymoma patients with and without paraneoplastic MG export mature naïve T cells. “Lymphocyte-rich” B2 thymomas, formerly cortical thymomas, reportedly exhibit higher percentages of mature naïve CD45^+^ T cells as compared to AB thymomas (mixed thymomas) or A thymomas (medullary thymomas) [56, 57]. Our present analysis revealed the highest absolute and relative lymphocyte counts in B1 and B2 thymomas, while neutrophil and platelet counts did not significantly differ among the WHO subtypes. Ströbel and coworkers demonstrated that paraneoplastic MG was highly associated with the capability of thymomas to produce and export naïve CD4^+^ T-cells [56]. Intratumorous thymopoiesis being prerequisite for MG development could explain the low incidence, or commonly the absence, of paraneoplastic MG in patients with TCs lacking the required organotypical features [1, 56, 58].

We also found that patients with TETs who were diagnosed with MG had significantly lower Fibrinogen serum concentrations compared to those without MG, while NLR and PLR did not differ between these two patient groups. Notably, after exclusion of TCs, Fibrinogen serum concentrations did not significantly differ in MG-positive compared to MG-negative thymomas (*p =* 0.051). Additionally, Masaoka-Koga tumor stage did not differ in accordance with MG status. These findings led us to postulate that differences in Fibrinogen serum concentrations were more likely caused by presence of TCs than by absence of paraneoplastic MG.

TETs are largely associated with good 10-year OS [8, 9, 30]. Even cases of tumor recurrence (reported in up to 30% of patients) have excellent 10-year OS rates of 75.0–90.5% after radical re-resection [9–11]. Since the specific tumor biology of TETs is characterized by slow tumor growth, relatively high tumor recurrence rates even decades after initial treatment, and excellent survival even after recurrent disease, it is recommended that patients receive life-long oncologic follow-up [11, 59]. At this time, the only recommended tool for oncologic follow-up is chest CT scanning, which is performed every 3–6 months for the first three years after tumor resection, and then annually thereafter [11]. There are currently no established serum biomarkers that can improve secondary and tertiary prevention. We recently demonstrated significantly increased CRP serum concentrations during the follow-up of patients who developed tumor recurrence. Based on this, we hypothesized that the detection of elevated CRP levels during oncologic surveillance might be helpful to predict tumor recurrence, and suggested CRP serum concentration monitoring might be included in the surveillance of patients with TETs [5].

Longitudinal analyses of Fibrinogen serum concentration, NLR, and PLR revealed that all three markers significantly increased in the immediate postoperative period. This was due to surgical stress that induces an acute phase response and corresponding increase of acute phase proteins [60]. Interestingly, within 6–12 months post-resection, NLR and PLR remained significantly elevated compared to preoperative values, while Fibrinogen serum levels returned to pretreatment concentrations. Stratifying patients based on tumor recurrence revealed that patients who developed tumor recurrence had significantly higher NLR and PLR values during follow-up respectively, 2.5-fold and 1.8-fold higher than in patients without recurrence. We assume that the increased NLR and PLR values in patients with tumor recurrence likely reflects re-induced tumor-related inflammation, as described above. NLR and PLR, respectively, predicted tumor recurrence with sensitivities of 80% and 100%, and negative predictive values of 96% and 100%. Based on these findings, we propose that NLR and PLR are accurate, cheap, easily measurable, and commonly available blood biomarkers that could help in the identification of patients with increased risk of recurrence during oncologic follow-up, potentially enabling early diagnosis and therapy.

We also previously demonstrated significantly increased CRP serum concentrations in cases of tumor recurrence, that high CRP serum levels predict worse FFR, and that CRP is a significant predictive marker for detection of tumor recurrence (*p =* 0.037; R^2^: 0.147).^7^ The predictive power was increased when CRP was used in combination with Fibrinogen (R^2^: 0.825), NLR (R^2^: 1.000), or PLR (R^2^: 0.726); however, the significance of each marker for predicting tumor recurrence was lost (Fibrinogen *p =* 0.352 and CRP *p =* 0.112; NLR *p =* 0.994 and CRP *p =* 0.991; PLR *p =* 0.726 and CRP *p =* 0.056). While NLR and CRP were significant predictive factors for detection of tumor recurrence, the combination of NLR with CRP or with other systemic biomarkers had no additive effect.

Our study has several weaknesses due to the mostly retrospective design and the single center experience, which limit our ability to draw conclusions. Nonetheless, the strengths of this study are the large number of patients having this rare disease, and the identification of the prognostic and diagnostic power of NLR, PLR, and Fibrinogen among patients with TETs.

Overall, we speculate that the observed increases of Fibrinogen, NLR, and PLR result from cancer-related inflammation, which is in line with our previous findings regarding cancer-related inflammation in TETs [7, 12, 13]. To our knowledge, serum biomarkers are not widely used in patients with TETs. We detected the highest pretreatment levels of Fibrinogen, NLR, and PLR in patients with more aggressive tumor behavior and higher tumor stage. High Fibrinogen, NLR, and PLR were also associated with worse outcome. During oncologic follow-up, NLR and PLR values were significantly increased in patients with tumor recurrence. We strongly believe that the systemic inflammatory parameters Fibrinogen, NLR, PLR, and CRP have substantial prognostic and diagnostic impact in patients with TETs, warranting future prospective multicenter studies.

## MATERIALS AND METHODS

### Study population

This study was conducted at the Department of Thoracic Surgery, Medical University of Vienna. We analyzed 122 patients with TETs who underwent surgical tumor resection between September 1999 and June 2015. The majority of patients (80.1%) had been treated within the past 10 years. All patients underwent preoperative chest CT scans for tumor staging, and some patients further received PET-CT scans or head and abdomen CT scans if advanced tumor stages were suspected. Primary surgical resection alone was performed in 45.9% of cases, while 54.1% of patients received multimodal treatment regimens. The median follow-up time was 30.8 months (range, 1 to 173.9 months). Oncologic follow-up involved periodic chest CT scans every 3 to 6 months for the first three years after surgery, followed by annual CT scans in accordance with the recommendations of the European Society of Thoracic Surgery (ESTS) [11].

The study also included 51 sex-matched (24 male, 27 female) and age-matched (54.6 ± 1.4 years) healthy volunteers as controls. These control participants underwent one-time measurements of Fibrinogen, NLR, and PLR. These values were used as reference values for analysis with the pretreatment Fibrinogen, NLR, and PLR measurements from patients with TETs.

### Outcome analysis

The main outcome parameters in this study were CSS and FFR. We evaluated outcome and recurrence following the recommendations of the International Thymic Malignancy Interest Group (ITMIG) [59]. Accordingly, CSS was calculated from the date of surgery to the date of death from TET, while unrelated deaths or deaths from unknown causes were censored. FFR was calculated in patients after radical tumor resection (R0), and was defined as the time from the date of surgery to the date of recurrence.

### Fibrinogen, NLR, and PLR measurement

Fibrinogen, platelets, and white blood cells, including neutrophils and lymphocytes, were measured during the routine preoperative work-up one day before surgery to exclude coagulation disorders/alterations or presence of acute infection. These parameters were also measured between 3 to 7 days postoperatively, and at 6 to 12 months after the initial therapy during oncologic follow-up. All analyses were performed by the department of laboratory medicine. Fibrinogen serum levels were evaluated using the Claus method as previously described [61]. NLR was calculated by dividing the relative neutrophil counts by the relative lymphocyte counts, whereas PLR was calculated by dividing the absolute platelet numbers by the absolute lymphocyte numbers. Preoperative, postoperative, and follow-up Fibrinogen serum levels were available from 112, 98, and 27 patients, respectively. Longitudinal NLR values were available in 101, 95, and 36 patients, respectively, while longitudinal PLR values were available for 96, 95, and 36 patients, respectively. Fibrinogen, NLR, and PLR measurements from 51 healthy sex- and age-matched volunteers served as controls for cross-sectional analysis.

### Immunohistochemistry

Immunohistochemistry was performed using the automated Ventana Benchmark^®^ platform (Ventana Medical Systems, Tuscon, AZ, USA). Staining was performed using monoclonal mouse anti-human CD45 (LCA; 2B11& PD7/26; Cell Marque, California, USA) and polyclonal rabbit anti-human Fibrinogen/FITC (Dako, Denmark). CD45 staining was performed to identify cells of the hematopoietic lineage. For CD45 staining, heat pre-treatment was performed for 56 min in Ultra cell conditioner Nr 1 buffer (Ultra CC1; pH 6). For Fibrinogen staining, Protease 1 was applied for 8 min. Samples were incubated with primary antibodies for 32 min, and then development was performed using the Ultraview Universal Detection DAB-kit following the manufacturer’s recommendations.

### Statistical methods

Statistical analyses were performed using SPSS software (version 22; IBM SPSS Inc., IL, USA). All data are reported in the Results section as mean ± standard error of the mean (SEM). We used the unpaired Student’s *t-*test and one-way ANOVA to compare the means of normally distributed variables with two or more than two groups, respectively. The Chi-square test was used to investigate associations between nominal variables. Survival analyses were performed using Kaplan-Meier analysis and the Log-rank test.

Univariable and multivariable Cox regression analyses were performed to evaluate the prognostic impacts of various clinical characteristics, including histology (TC vs. Thymoma), Masaoka-Koga tumor stage (I–II vs. III– IV), Fibrinogen (high vs. low), NLR (high vs. low), and PLR (high vs. low)—on CSS and FFR. We plotted the receiver operating characteristic (ROC) curve calculated the Youden Index to identify an optimal Fibrinogen cut-off value of 452.5 mg/dL to differentiate between the high and low Fibrinogen subgroups. We used an empiric cut-off value of 4.0 to differentiate between high and low NLR cohorts [32, 62], and a median PLR of 136.5 to determine high and low PLR cohorts. We performed binary logistic regression analysis to evaluate the predictive values of Fibrinogen, NLR, and PLR for detection of tumor recurrence within follow-up. R^2^ values are indicated as markers for the goodness-of-fit of our models.
